# Genome-wide analysis of the SCAMPs gene family of soybean and functional identification of *GmSCAMP5* in salt tolerance

**DOI:** 10.1186/s12870-023-04649-2

**Published:** 2023-12-08

**Authors:** Min Wang, Chuanrong He, Guangcheng Shi, Qiukai Yin, Hanyue Zhang, Wanmin Yang, Aiqin Yue, Lixiang Wang, Weijun Du

**Affiliations:** 1 College of Agriculture, Shanxi Agricultural University, Taigu, 030801 China; 2 Houji laboratory in Shanxi Province, Shanxi Agricultural University, Taigu, 030801 China; 3 Department of Biological Science and Technology, Jinzhong University, Yuci, 030619 China

**Keywords:** Secretory carrier protein (SCAMP), Soybean, Salt tolerance, Na^+^/K^+^, Salt stress-related genes

## Abstract

The effect of salt damage on plants is mainly caused by the toxic effect of Na^+^. Studies showed that the secretory carrier membrane proteins were associated with the Na^+^ transport. However, the salt tolerance mechanism of secretory carrier protein (SCAMP) in soybean was yet to be defined. In this study, ten potential *SCAMP* genes distributed in seven soybean chromosomes were identified in the soybean genome. The phylogenetic tree of SCAMP domain sequences of several plants can divide SCAMPs into two groups. Most *GmSCAMPs* genes contained multiple Box4, MYB and MYC *cis*-elements indicated they may respond to abiotic stresses. We found that *GmSCAMP1*, *GmSCAMP2* and *GmSCAMP4* expressed in several tissues and *GmSCAMP5* was significantly induced by salt stress. *GmSCAMP5* showed the same expression patterns under NaCl treatment in salt-tolerant and salt-sensitive soybean varieties, but the induced time of *GmSCAMP5* in salt-tolerant variety was earlier than that of salt-sensitive variety. To further study the effect of *GmSCAMP5* on the salt tolerance of soybean plants, compared to *GmSCAMP5*-RNAi and EV-Control plants, *GmSCAMP5*-OE had less wilted leave and higher SPAD value. Compared to empty vector control, less trypan blue staining was observed in *GmSCAMP5*-OE leaves while more staining in *GmSCAMP5-*RNAi leaves. The Na^+^ of *GmSCAMP5*-RNAi plants leaves under NaCl stress were significantly higher than that in EV-Control plants, while significantly lower Na^+^ in *GmSCAMP5*-OE plants than in that EV-Control plants. The contents of leaves K^+^ of *GmSCAMP5*-RNAi, EV-Control, and *GmSCAMP5*-OE plants under NaCl stress were opposite to that of leaves Na^+^ content. Finally, salt stress-related genes *NHX1*, *CLC1*, *TIP1*, *SOD1*, and *SOS1* in transformed hairy root changed significantly compared with the empty control. The research will provide novel information for study the molecular regulation mechanism of soybean salt tolerance.

**Supplementary Information:**

The online version contains supplementary material available at 10.1186/s12870-023-04649-2.

## Backgrounds

Soil salinization has become a worldwide problem. When plants are grown in saline-alkali land, the water potential decreases and an ionic imbalance occurs, which is toxic to plants. This can cause plants to stop growing and even lead to their death [1–3]. The severe restriction of crop growth and agricultural development due to soil salinization highlights the significance of improving the utilization of saline-alkali land for sustainable agricultural development. Soybean, as an important oil and economic crop, has witnessed an increasing demand over the years. Cultivated soybean is considered to have moderate salt tolerance [4], but it suffers from salt injury when the soil salinity exceeds 5 dS.m^− 1^, and no harvest is possible under higher salt concentrations [5]. Therefore, studying the mechanisms of salt tolerance and breeding salt-tolerant soybean varieties is crucial for improving soybean yield and quality.

Osmotic stress [1, 2], plasma membrane damage [3], ion imbalance [6, 7], and metabolic disorder [8] are the primary causes of salt stress. Among these, ion balance is the key issue in understanding salt tolerance mechanisms. Research has shown that salt damage in soybeans is caused by various ions. The accumulation of these ions leads to damage such as leaf loss, withering, and a decline in plant photosynthetic capacity. Sodium (Na^+^) is the main lethal ion, and the plant’s susceptibility to salt damage is closely related to the Na^+^ content in its tissues [9]. Studies have demonstrated that plant cells have mechanisms to expel or transfer Na^+^ to inactive metabolic zones when it enters the cells. Na^+^/H^+^ transporters in vacuoles play a crucial role in transporting Na^+^ to vacuoles, thereby reducing its concentration in the cells [10]. Soybean possesses a chloride (Cl^−^) discharge mechanism that is regulated by a single gene. When the Cl^−^ content in each tissue of the plant reaches a certain level, the excess ions can be effluxed from the tissue, thereby minimizing the damage caused by ions to soybean. This distribution and efflux of salt ions primarily occur through ion transporters, which are present in the cytoplasmic membrane. These ion transporters selectively reduce ion toxicity by transporting the relevant salt ions. Among the extensively studied proteins are the Na^+^/K^+^ and Na^+^/H^+^ conversion proteins. SOS1, a Na^+^/H^+^ antiporter situated in the cell membrane and associated with salt tolerance, facilitates the transport of Na^+^ to the vacuole, thereby reducing ion toxicity. In a study conducted by Nie et al. (2015), it was demonstrated that the ion transporter GmSOS1 significantly enhanced the seed germination rate of mutants under salt stress [11]. Another important protein is NHX, a Na^+^/H^+^ reverse transporter located on the vacuolar membrane, which aids in compartmentalizing Na^+^ into the vacuole [12, 13]. Previous studies have demonstrated that the Na^+^/H^+^ transporter GmNHX1 and chloride channel protein GmCLC1 in soybean can significantly enhance the tolerance of tobacco cells to salt stress [14, 15]. The overexpression of *TaNHX2* has been found to enhance salt tolerance in both ‘composite’ and whole transgenic soybean plants [13], while the overexpression of NHX1 has been shown to improve salt tolerance in whole transgenic soybean plants [16]. Similar conclusions have been drawn for other crops as well. For instance, the overexpression of the K^+^/Na^+^ transporter *TaNHX3* gene in wheat has been found to enhance salt tolerance in tobacco [17], and the *TaNHX2* gene has been shown to improve salt tolerance in transgenic pepper plants [18]. Additionally, Chen et al. (2014) discovered that the soybean potassium ion transporter *GmHKT1* gene also enhances tobacco salt tolerance [19]. Overall, these findings indicate the significant importance of antitransporters in enhancing salt tolerance in soybean.

Previous studies discovered NHE7 could interact with SCAMP2 in human and NHE5 can interact with SCAMP2 in mammals [20, 21]. These findings suggest that the function of Na^+^/H^+^ reverse transporters is influenced by the secretion of the carrier membrane protein SCAMP. SCAMPs are IV-type membrane proteins that participate in the endocytosis pathway of cells and are located in the plasma membrane, primary plastids, or the reverse Golgi [22]. Bai et al. (2020) showed that the expression of the *AtSCAMP* genes from *Arabidopsis thaliana* was up-regulated in response to salt stress [23]. However, there is currently no research on the functional and mechanical effects of the SCAMP in soybean under salt stress.

In this study, we utilized a systemic bioinformatics approach to identify and analyze the soybean SCAMP protein family. The relative expression level of the *GmSCAMP5* gene was compared in salt-tolerant and salt-sensitive soybean varieties, and the gene was overexpressed in soybean hairy roots. The study investigated the salt tolerance mechanism of *GmSCAMP5* by analyzing the activities of Na^+^ and K^+^ content in the root and leaf, as well as the expression levels of potential ion transport related genes in *GmSCAMP5*-overexpressing soybean hairy roots under salt stress. The findings demonstrate the positive role of *GmSCAMP5* in ion transport under salt stress and provide new insights into the role of SCAMP in response to salt stress and the underlying mechanisms.

## Results

### Identification and characterization of *GmSCAMP* Gene family members

Ten potential *SCAMP* genes were identified in the soybean genome and named *GmSCAMP1* to *GmSCAMP10* according to their position on seven chromosomes (Table S1). The length of the GmSCAMP proteins ranged from 247 (GmSCAMP3) to 310 amino acids (GmSCAMP10). The smallest protein molecular weight (MW) is 27751.43 Da (for GmSCAMP3), while the largest protein MW is 34658.96 Da (for GmSCAMP10). The isoelectric point (pI) of the proteins ranged from 6.61 (GmSCAMP10) to 8.86 (GmSCAMP3), indicating that all SCAMPs were neutral amino acids. The hydropathicity values ranged from 0.09 to 0.23, which were greater than 0, suggesting that these proteins were hydrophobic. Furthermore, each GmSCAMP protein contained 4 transmembrane domains. Soybean SCAMP proteins possess a shared SCAMP conserved domain (Figure S1), indicating that each SCAMP protein contains only one SCAMP conserved domain.

### Chromosomal distribution of *GmSCAMP* family members

The GmSCAMPs’ physical location map was created using the physical location data from the soybean genome. The 10 SCAMP genes were unevenly dispersed across the 7 soybean chromosomes (Figure S2). Chromosome 13 had the highest number of GmSCAMP genes (3 genes), trailed by Chromosome 12 (2 genes). Chromosomes 1, 6, 7, 9, 15 contained only 1 gene each. Significantly, we detected that most SCAMPs were located at the chromosomal ends.

### Phylogenetic analysis of *GmSCAMP* Gene family

**Fig. 1:**
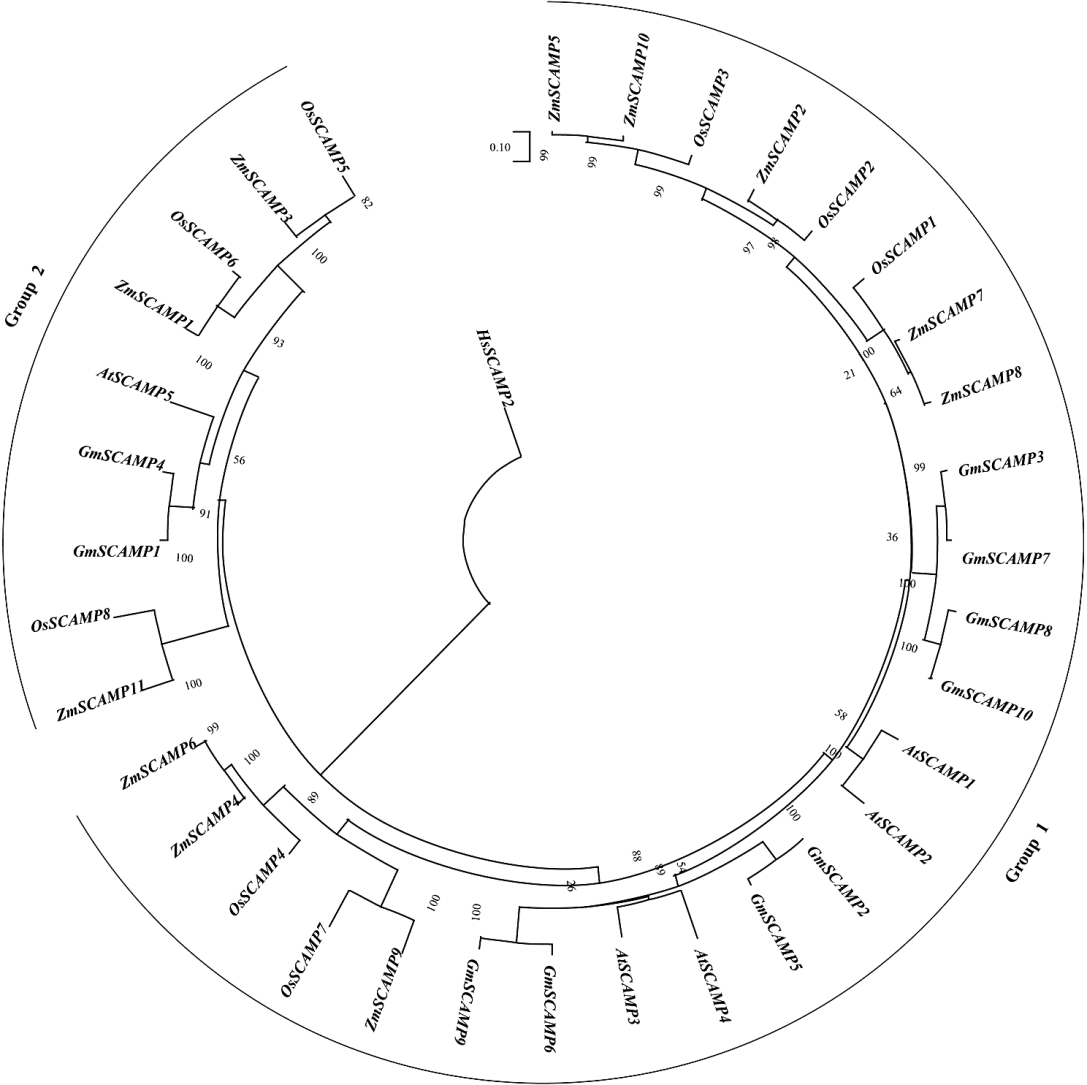
Phylogenetic tree of SCAMP proteins in soybean, *Arabidopsis thaliana*, maize and rice. The domain amino acid sequences of SCAMP proteins were aligned by Clustal W. The phylogenetic tree was constructed and was rooted by using the human SCAMP2 as the outgroup using the Neighbor-Joining method in MEGA 7.0. The numbers next to the branches are bootstrap values (for 1000 replicates, given as a percentage). Protein sequences of soybean, *Arabidopsis thaliana*, maize and rice were divided into two Groups, namely, Group 1 and Group 2 in an original tree in a radiation topology. 4 species have at least one representative in both SCAMP groups

To investigate the phylogenetic relationships between *SCAMP* genes in soybean, we constructed phylogenetic trees using the alignment of SCAMP domain amino acid sequences derived from soybean (10), *Arabidopsis* (5), maize (11), and rice (8), and was rooted by using the human SCAMP2 as the outgroup *via* the Neighbor-Joining (NJ) method in MEGA7.0 (Fig. 1). 4 species SCAMPs form a clade distinctive from human SCAMP2 proteins. The resultant tree depicted the *SCAMP* genes divided into two groups. Group 1 contained eight soybean, four *Arabidopsis*, eight maize, and five rice SCAMP members respectively. Group 2 consisted of two members of the soybean SCAMP family, as well as one *Arabidopsis*, three maize, and three rice SCAMP family members respectively. The ratio of Group 1 to Group 2 among soybean SCAMP family members was 4:1, which is consistent with that of *Arabidopsis*. It showed that 4 species have at least one representative SCAMP family member in both SCAMP groups.

### Gene structure and motif composition of soybean *SCAMP* Genes

To determine the differences in gene composition, a comparative analysis of exon and intron structures was performed on ten soybean *SCAMP* genes (Figure S3). The majority of *SCAMP* gene sequences consisted of exons, introns and UTR. Notably, only *GmSCAMP8* and *GmSCAMP10* lack UTR within ten *SCAMP* gene sequences. The range of exons across all *SCAMP* genes was between 11 and 13, with most *SCAMP* genes in the same group or subgroup demonstrating conserved exons. The range of introns across all *SCAMP* genes was between 11 and 14. The positions of the exons varied among the different members of the *SCAMP* gene, as did the positions of the introns.

The conserved motifs in the ten GmSCAMP proteins was analyzed using the MEME website. A total of 10 conserved motifs were identified, designated as motif1-10 (Figure S4). Each SCAMP protein contained 5 to 8 motifs. Motif1, motif2, motif3, and motif4 were present in all SCAMP proteins, demonstrating their high levels of conservation. SCAMPs in the same group shared similar motifs, as was observed in GmSCAMP1 and GmSCAMP4.

### *Cis*-acting elements located in promoters of *GmSCAMP* genes

**Fig. 2:**
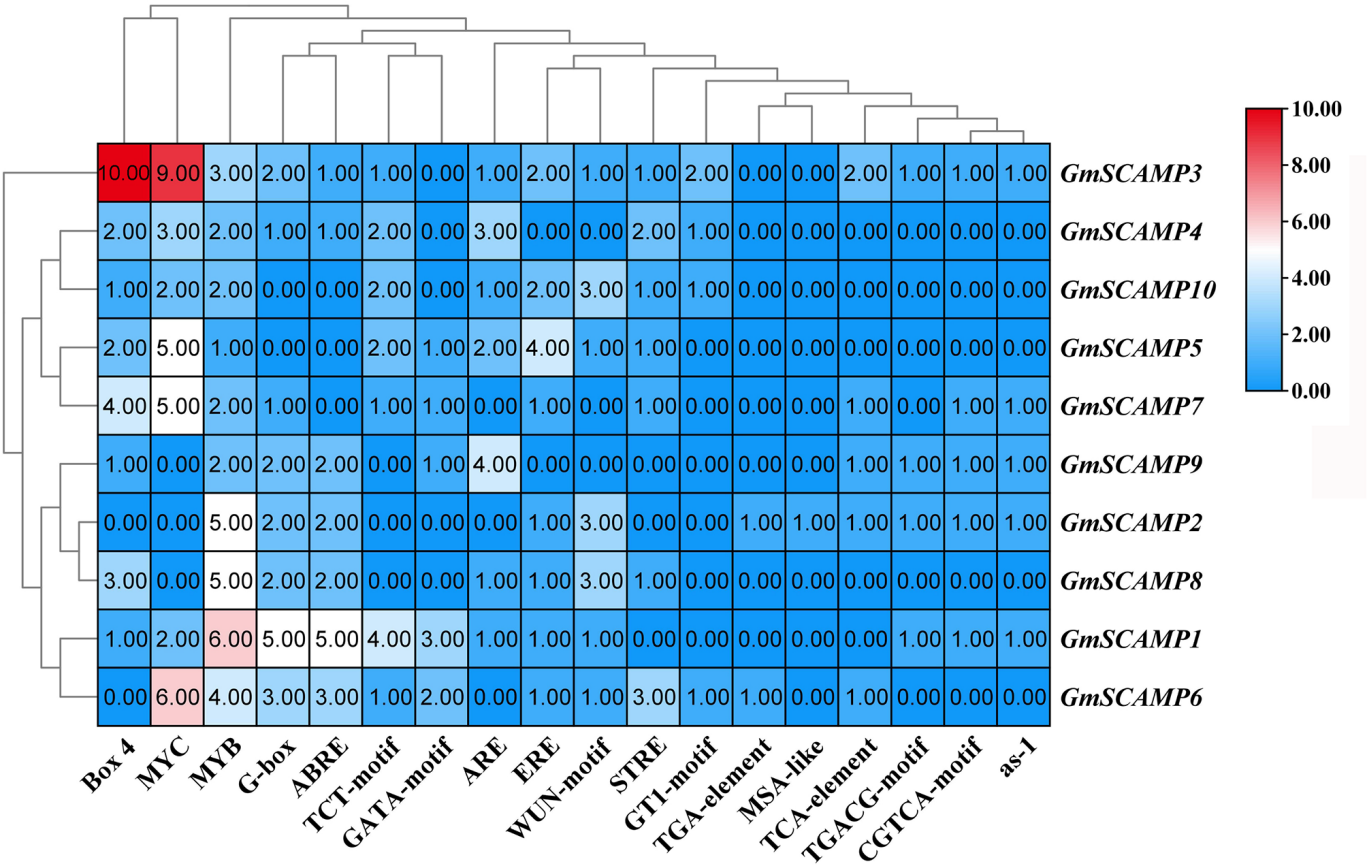
Promoter stress-related *cis*-acting elements of soybean *SCAMP* genes. Promoter sequences (1500 bp upstream of start codon site) of *GmSCAMPs* were submitted to Plant CARE database to identify *cis*-acting elements. Correlation is represented by the color: red, higher correlation; blue, lower correlation. MYC, MYB, G-box and STRE are stress response elements; Box 4 is hormone, drought and low temperature response element; ABRE is abscisic acid response element; TCT-Motif is hormone response element; GATA-Motif is drought response factor; ARE is ethylene response element; ERE and GT1-motif are light and hormone response element; WUN-motif is mechanical injury response elements; TGA-element is temperature response element; TGA-element is auxin responsive element; MSA-like, TCA-element: temperature response factor; TGACG-Motif and CGTCA-motif: methyl jasmonate response elements; as-1 is disease-resistant response elements

To explore the gene expression pattern, firstly, *cis*-element analysis was conducted in the 1500 bp region upstream of the start codon in the promoter of each *GmSCAMPs* (Fig. 2). Several essential *cis*-elements were identified, including biotic, abiotic stress-responsive elements, as well as hormone-responsive elements. Most *GmSCAMPs* contained a considerable number of Box4, MYB, and MYC and hence indicate their potential response to various abiotic stresses, including hormone, drought, and cold. These findings suggest that *GmSCAMPs* have a significant regulatory effect to withstand adversity stresses.

### Expression patterns of *GmSCAMP* Genes

Through further exploration of RNA-seq data and relevant literature, it was discovered that the expression of *SCAMP* genes in various tissues is not specific to any particular tissue. In the case of whole seeds at different stages of development (globular, heart, cotyledon, early-maturation, mid-maturation, late-maturation, dry), as well as vegetative tissues (leaves, roots, stems, seedlings) and reproductive tissues (floral buds), *GmSCAMP1*, *GmSCAMP2*, *GmSCAMP3*, *GmSCAMP4*, *GmSCAMP5*, and *GmSCAMP7* exhibited relatively high expression across different tissues. On the other hand, *GmSCAMP6*, *GmSCAMP8*, *GmSCAMP9*, and *GmSCAMP10* displayed overall weak basal expression (Figure S5). In *Arabidopsis*, *AtSCAMP5* demonstrated high and widespread temporal and spatial expression patterns in most tissues, whereas *AtSCAMP2* and *AtSCAMP3* exhibited overall weak basal expression. Similarly, the expression of *SCAMP* genes in different tissues of *Arabidopsis* was not specific to any particular tissue.

**Fig. 3:**
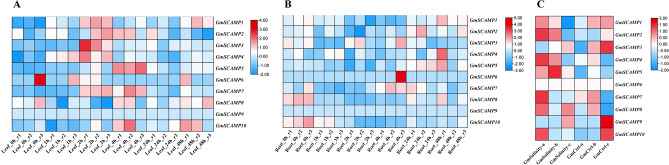
Expression profiles of *GmSCAMP* genes under salt stress. The expression abundance of each transcript is represented by the color: red, higher expression; blue, lower expression. **(A, B)** Expression levels of leaf **(A)** and root **(B)** in different time period under salt stress are shown: 0 h, 1 h, 2 h, 4 h, 24 h, 48 h. **(C)** Expression levels of root hair cells under salt stress are shown

Under salt stress, *GmSCAMP3* in the leaves expressed highest at 2 h, while *GmSCAMP5* exhibited highest expression levels at 4 h (Fig. 3A). In the roots, *GmSCAMP5* displayed increased expression levels during the later stage of salt stress (Fig. 3B). In addition, *GmSCAMP2*, *GmSCAMP4*, and *GmSCAMP5* exhibited higher expression levels in root hair cells under high salinity conditions (Fig. 3C). It was observed that *SCAMP* genes were expressed in the leaves, roots, and root hair cells under salt stress, with *GmSCAMP5* showing a significantly high expression level. The qRT-PCR results of this study indicated that *GmSCAMP5* exhibited high expression levels in the leaves of soybean during early salt stress (Fig. 3). In conclusion, it can be inferred that *GmSCAMP*5 may be influenced by salt stress.

### Expression analysis of *GmSCAMPs* under salt stress

**Fig. 4:**
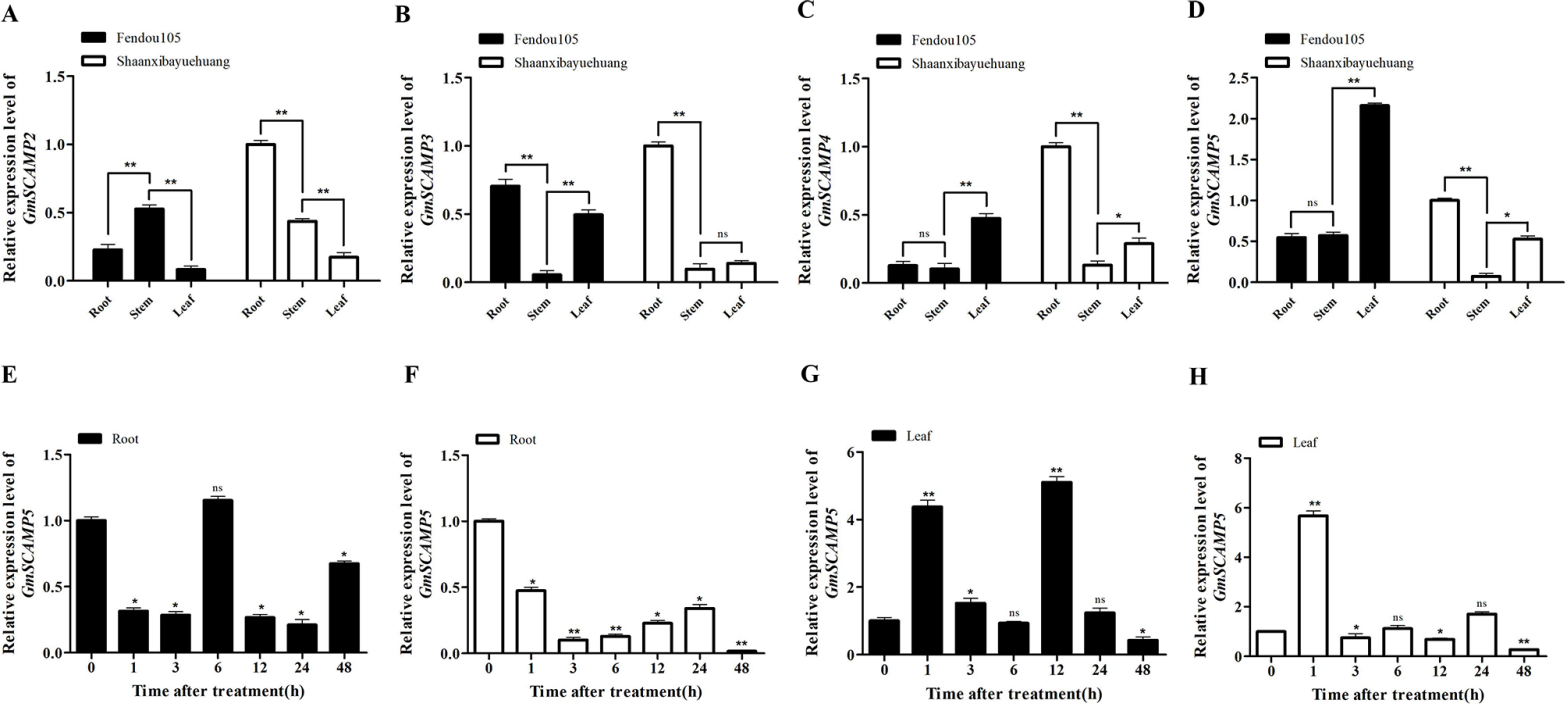
Expression pattern of soybean *GmSCAMPs* in salt-tolerance materials and salt- sensitive materials. **A-D** shown the relative expression level of *GmSCAMP2, GmSCAMP3, GmSCAMP4, GmSCAMP5* in leaf, stem and root at 12 h of salt-tolerant materials and salt-resistant materials, respectively. **E-H** shown the relative expression level of *GmSCAMP5* in root and leaf of salt-tolerant materials and salt-sensitive materials at 0, 1, 3, 6, 12, 24, 48 h. *GmCYP2* was used as an internal control. *, *P* < 0.05; **, *P* < 0.01; ns, no significant difference

To further investigate the potential function of *GmSCAMP2*, *GmSCAMP3*, *GmSCAMP4*, and *GmSCAMP5* in soybean salt-tolerance, we examined their temporal and spatial expression patterns using qRT-PCR. We observed that these genes exhibited different tissue expression patterns in salt-tolerant materials, but similar patterns in salt-sensitive materials (Fig. 4A-4D). Specifically, in the salt-tolerant material ‘Fendou105’, *GmSCAMP2* was highly expressed in the stems, *GmSCAMP3* in the roots and leaves, *GmSCAMP4* and *GmSCAMP5* in the leaves. In the salt-sensitive material ‘Shaanxibayuehuang’, *GmSCAMP2*, *GmSCAMP3*, *GmSCAMP4*, and *GmSCAMP5* were highly expressed in the roots. Notably, compared to other genes, the expression level of *GmSCAMP5* in ‘Fendou105’ was higher than in ‘Shaanxibayuehuang’ in the most of tissues at 12 h. Further analysis revealed that the expression of *GmSCAMP5* in the roots of both materials significantly decreased, except for the roots of ‘Fendou105’ at 6 h (Fig. 4E and F). Additionally, *GmSCAMP5* in the leaves of ‘Fendou105’ at 1 h, 3 h, and 12 h showed higher expression compared to the control (0 h) (Fig. 4G), while the expression of the gene in the leaves of ‘Shaanxibayuehuang’ showed higher expression at 1 h compared to the control (0 h) (Fig. 4H). These results suggest *GmSCAMP5* may play an important role in soybean salt stress responses.

### *GmSCAMP5* improves salt tolerance in soybean hairy roots

**Fig. 5:**
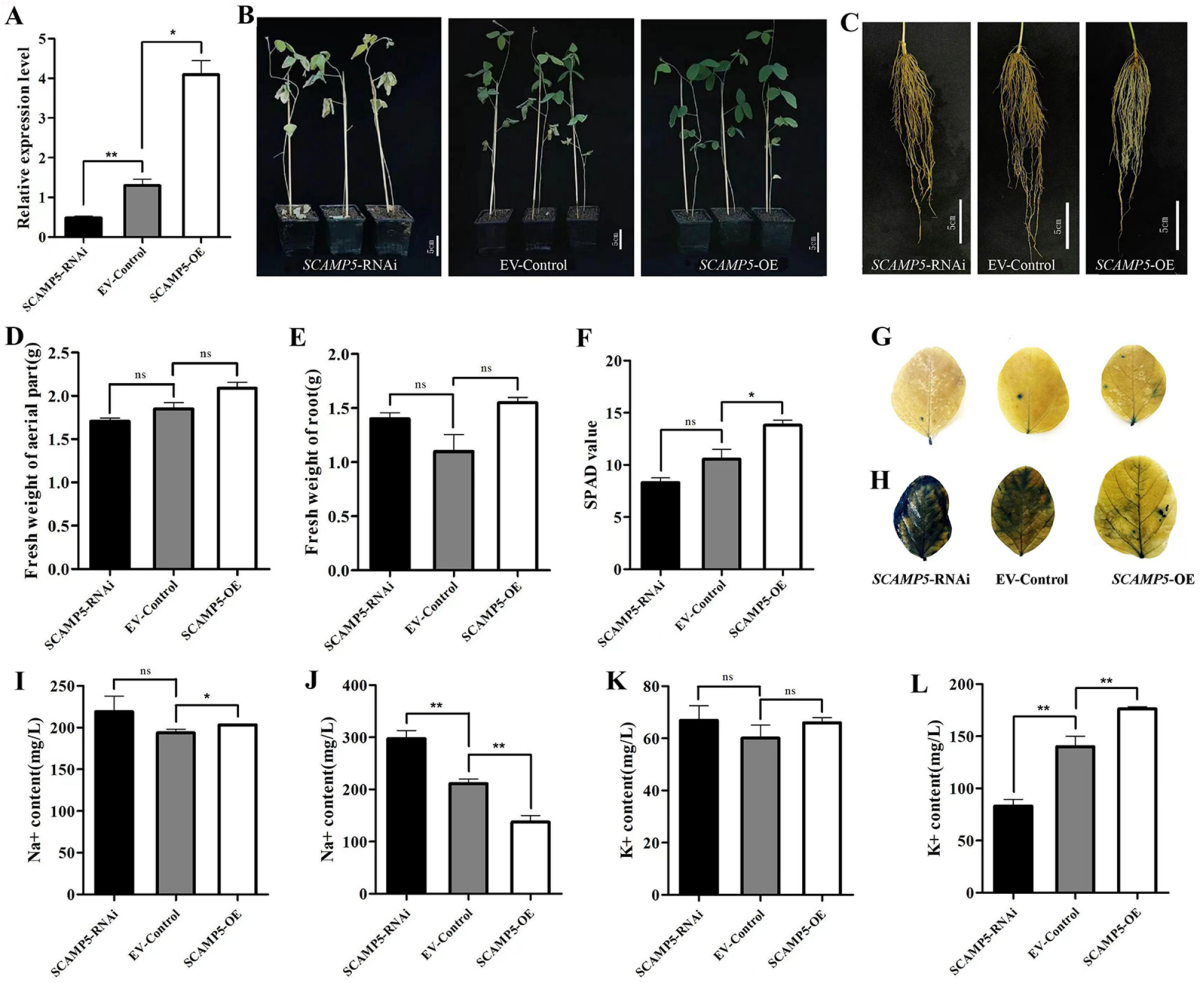
Analysis of the function of soybean *GmSCAMP5* under salt stress. **A** Relative expression analysis of positive hairy roots (**p < 0.01, *p < 0.05). **B** Phenotypes of hairy root transformation soybean plants, include *SCAMP5*-RNAi, EV-Control, and *SCAMP5*-OE under salt stress. **C** The roots of hairy root transformation soybean plants, include* SCAMP5*-RNAi, EV-Control, and *SCAMP5*-OE under salt stress. **D, E** Fresh weight of aerial part**(D)** and root**(E)** in hairy root transformation soybean plants, include *SCAMP5*-RNAi, EV-Control, and *SCAMP5*-OE under salt stress. **F**, SPAD value in hairy root transformation soybean plants, include *SCAMP5*-RNAi, EV-Control, and *SCAMP5*-OE under salt stress. **G, H** Trypan blue staining of leaves of hairy root transformation soybean plants, include *SCAMP5*-RNAi, EV-Control, and *SCAMP5*-OE under normal conditions**(G)** and salt stress**(H)**, the dead cells can be strained, but living cells cannot. **I-L** Na^+^ content of root**(I)** and leaf**(J)**, and K^+^ content of root**(K)** and leaf**(L)** in hairy root transformation soybean plants, include *SCAMP5*-RNAi, EV-Control, and* SCAMP5*-OE under salt stress

To further verify the function of *GmSCAMP5* in vivo, we constructed *GmSCAMP5*-OE, EV, and *GmSCAMP5*-RNAi soybean hairy roots (Figure S6), Positive transgenic hairy roots were selected and further confirmed by qRT-PCR assay. qRT-PCR analysis showed that the expression level of *GmSCAMP5* in OE transgenic hairy roots was much higher than that in the EV-transformed control, while the expression level of *GmSCAMP5* in RNAi transgenic hairy roots was lower than that in the EV (Fig. 5A). Under NaCl stress, the *GmSCAMP5*-OE transgenic plants and their roots displayed almost the same size as the *GmSCAMP5*-RNAi and EV-Control did (Fig. 5B, C). However, there were noticeable differences in the degree of leaf yellowing and wilting among the *GmSCAMP5*-RNAi, EV-Control, and *GmSCAMP5*-OE plants (Fig. 5B). On *GmSCAMP5*-RNAi plants, all leaves wilted and yellowed after NaCl treatment. On EV-Control plants, the lower leaves wilted while the upper leaves were basically unaffected. On *GmSCAMP5*-OE plants, all leaves were largely unaffected by salt damage. We analyzed the fresh weight of the aerial part and roots, we found that there were no significant differences in these indexes between the *GmSCAMP5*-RNAi, EV-Control, and *GmSCAMP5*-OE plants under NaCl stress (Fig. 5D and E).

The chlorophyll content is measured using the SPAD value, which is positively correlated with chlorophyll. Chlorophyll plays a crucial role in photosynthesis in plants. In this study, the SPAD value was used to assess the salt tolerance of genetically modified soybean plants with transgenic hairy roots, including *SCAMP5*-RNAi, EV-Control, and *SCAMP5*-OE, under salt stress. The SPAD value in the leaves of *GmSCAMP5*-OE plants was significantly higher than that in EV-Control plants under NaCl stress. Furthermore, the SPAD value in the leaves of EV-Control plants was higher than that in *GmSCAMP5*-RNAi plants under NaCl stress, although the difference was not statistically significant. (Fig. 5F**)**.

Trypan blue staining was used to observe cell activity in leaves of *GmSCAMP5*-RNAi, EV-Control, and *GmSCAMP5*-OE composite plants. No significant difference in trypan blue staining was observed under normal growth conditions (Fig. 5G). However, under salt treatment conditions, *GmSCAMP5*-OE showed less staining than EV-Control. In contrast, *GmSCAMP5*-RNAi leaves exhibited more intense staining than EV-Control leaves (Fig. 5H). The results indicate that cell membrane integrity and stability were significantly higher in leaves of *GmSCAMP5*-OE plants than in leaves of EV-Control and *GmSCAMP5*-RNAi plants.

To further investigate the role of *GmSCAMP5* in ion transport, we analyzed the Na^+^ and K^+^ content in the leaves and roots. We found no significant differences in Na^+^ content in roots between the *GmSCAMP5*-RNAi, EV-Control, and *GmSCAMP5*-OE plants under NaCl stress. The Na^+^ content in the leaves of *GmSCAMP5*-RNAi plants was significantly higher than that in EV-Control plants under NaCl stress. Conversely, the leaves of *GmSCAMP5*-OE plants had significantly lower Na^+^ content than those in EV-Control plants (Fig. 5I J). There were no notable differences in K^+^ content in the roots of *GmSCAMP5*-RNAi, EV-Control, and *GmSCAMP5*-OE plants exposed to NaCl stress. The potassium content of leaves in *GmSCAMP5*-OE plants subjected to NaCl stress was significantly higher than that in EV-Control plants. Conversely, the potassium content of leaves in *GmSCAMP5*-RNAi plants was significantly lower than that in EV-Control plants (Fig. 5K L).

### Mechanism of *GmSCAMP5* in regulating salt soybean tolerance

**Fig. 6:**
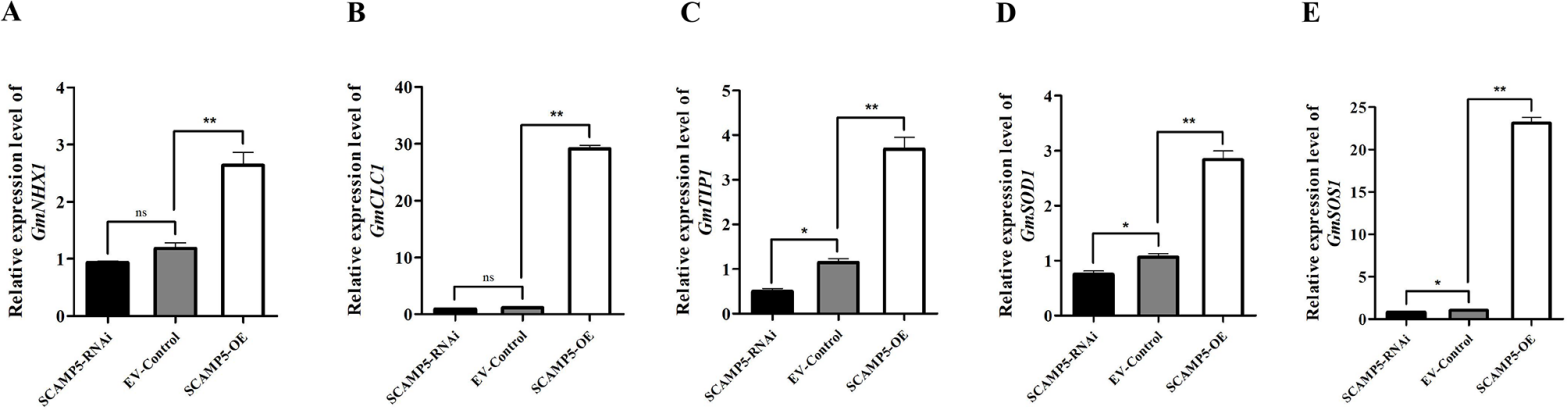
Expression levels of salt tolerance related genes in hairy root transformation soybean plants. qRT-PCR analysis of *GmNHX1***(A)**, *GmCLC1***(B)**, *GmTIP1***(C)**, *GmSOD1***(D)**, and *GmSOS1***(E)** in in hairy root transformation soybean plants, include *SCAMP5*-RNAi, EV-Control, and *SCAMP5*-OE under salt stress. The transcript amounts in each sample were normalized to those of *GmCYP2* (Student’s t-test; *p < 0.05, **p < 0.01)

To explore the mechanism *GmSCAMP5* in regulate soybean salt stress, the maker genes expression pattern of soybean salt stress response were checked. Under salt treatment conditions, the relative expression levels of *GmNHX1*, *GmCLC1*, *GmTIP1*, *GmSOD1* and *GmSOS1* were significantly higher in *GmSCAMP5*-OE compared to the EV-Control. On the other hand, the relative expression levels of *GmNHX1*, *GmCLC1*, *GmTIP1*, *GmSOD1* and *GmSOS1* were decreased in *GmSCAMP5*-RNAi compared to the EV-Control, with a significant decrease in the expression levels of *GmTIP1*, *GmSOD1* and *GmSOS1* (Fig. 6). These findings suggest that *GmSCAMP5* may regulate soybean salt stress by inducing the expression of salt-tolerance related genes, thereby enhancing the salt tolerance of soybean and reducing the degree of salt damage in plants.

## Discussion

A total of 10 *SCAMP* genes were identified in soybean, while 5 *AtSCAMP* genes were identified in Arabidopsis, this gene family enlargement may result in the specific role in soybean *SCAMP* gene family [23]. In a previous study [22], a phylogram was created based on the amino acid sequences of putative plant SCAMPs, with a variety of animal SCAMPs as outgroups. The study found that angiosperm SCAMPs can be divided into two clusters, with a bootstrap support of over 60%. Each cluster contains representatives from all angiosperm taxa (including monocots and dicots) except Manihot esculenta. This suggests a functional differentiation of SCAMPs in angiosperm lineages. In our study, we constructed phylogenetic trees using SCAMP domain amino acid sequences from soybean, two monocotyledons (rice and maize), and one dicotyledon (*Arabidopsis*), with human SCAMP2 as outgroups. The resulting phylogenetic trees showed that the SCAMPs from the four species (soybean, *Arabidopsis*, maize, and rice) formed a distinct clade separate from human SCAMP2 proteins. These findings are consistent with previous reports. GmSCAMP, a SCAMP protein found in soybean, has a highly conserved SCAMP domain and a small protein length ranging from 137 to 172 amino acids.The number of introns and exons in *GmSCAMP* gene family members varied significantly, suggesting potential changes during evolution. The promoter regions of *GmSCAMPs* contain numerous stress-related *cis*-acting elements, indicating their possible involvement in stress responses.

The expression patterns of plant genes are closely related to their functions. It was observed that the expression of *SCAMP* genes in different tissues was not tissue-specific (Figure S5). Among the *SCAMP* genes, *GmSCAMP1*, *GmSCAMP2*, *GmSCAMP3*, *GmSCAMP4*, *GmSCAMP5*, and *GmSCAMP7* showed relatively high expression across different tissues, while *GmSCAMP6*, *GmSCAMP8*, *GmSCAMP9*, and *GmSCAMP10* had weak basal expression. In *Arabidopsis*, *AtSCAMP5* exhibited high and ubiquitous temporal and spatial expression patterns in most tissues, whereas *AtSCAMP2* and *AtSCAMP3* showed overall weak basal expression [22]. Similarly, the expression of *SCAMP* genes in different tissues was not tissue-specific in Arabidopsis.

Previous studies have only reported on the role of *SCAMP* genes in NaCl stress. Research on *Arabidopsis thaliana* has shown that *AtSCAMP1*-*5* genes are up-regulated in the roots and leaves under NaCl stress, with significantly higher expression levels in the leaves compared to the roots. These findings suggest that *AtSCAMPs* genes respond to NaCl stress in *Arabidopsis thaliana* [23]. However, no studies have been found on *GmSCAMPs* in soybean under NaCl stress. The expression levels of *SCAMPs* under salt stress were analyzed using RNA-seq data. Some RNA-seq data revealed highly expression of *GmSCAMP2* and *GmSCAMP5* in nodules (extracted from NCBI-SRA database: PRJNA551959). This provides valuable gene resources for investigating the mechanism of symbiotic nitrogen fixation in soybean and the efficiency of nitrogen fixation in soybean nodules. Furthermore, RNA-seq data indicated that *GmSCAMP2*, *GmSCAMP4*, and *GmSCAMP5* exhibited higher expression levels in root hair cells under high salinity conditions (Fig. 3C). As roots are the organs directly contact with the soil, they are more susceptible to salt stress. Therefore, we do not exclude the possibility of these genes in regulating soybean nodulation, but studying the expression and function of these genes in the roots is crucial in understanding salt tolerance.

In our previous study, Shi et al. (2022) [24] identified two varieties with different salt tolerance. We selected this two materials for gene expression analysis. We found under 150mM NaCl conditions, ‘Fendou105’ exhibited significantly higher dry weight, plant height, and root length compared to ‘Shaanxibayuehuang’. The leaves and stems of ‘Fendou 105’ contained less Na^+^ and Cl^−^ than ‘Shanxibayuehang’, while the roots of ‘Fendou 105’ contained higher Na^+^ and Cl^−^. The SPAD of ‘Fendou105’ significantly increased under 150mM NaCl conditions, while that of ‘Shanxibayuehang’ was decreased. These findings indicate that these two materials possess different salt response pattern, including ion transport and photosynthesis characteristics. Therefore, studying the expression level of SCAMPs under salt stress in these two soybean varieties is important. Many studies investigated salt tolerance in soybean using RNA-seq, focusing on either the stage of fully developed true leaves [25] or fully expanded first trifoliate leaves [26–28]. In this study, we utilized RNA-seq data from primary leaves, which are also known as true leaves. The salt-sensitive materials used for qRT-PCR analysis were highly susceptible to salt stress, and subjecting them to salt stress treatment at the primary leaves expanded stage would result in plant death. To ensure that the plants experienced damage but remained alive, we selected the period of first trifoliate leaves expansion for treatment.

*GmSCAMP5* in the leaf exhibited the highest expression levels at 4 h. When comparing RNA-seq data with qRT-PCR data, it was observed that the expression peak of *GmSCAMP5* in leaves was different. However, it was consistently induced early period during salt stress. Additionally, it was discovered that the duration of induced expression was longer in salt-tolerant materials compared to salt-sensitive materials. In RNA-seq data *GmSCAMP5* displayed increased expression levels in the roots during significantly decreased, except for the roots of ‘Fendou105’ at 6 h in this study. However, the expression of *GmSCAMP5* in the roots of ‘Fendou105’ increased at 48 h compared to 12 h and 24 h. In contrast, the expression of *GmSCAMP5* in the roots of ‘Shaanxibayuehuang’ decreased at 48 h compared to 1 to 24 h. The expression pattern of *GmSCAMP5* in the roots of salt-tolerant materials was similar to that observed in the RNA-seq data, suggesting that the expression of *GmSCAMP5* in the roots may be induced in salt-tolerant materials during the later stages of salt stress. It is evident that the expression of *GmSCAMP5* in the roots of salt-sensitive materials was down-regulated under salt stress, which could be one of the reasons for the difference in salt tolerance between the two materials.

The association of the *SCAMP* gene family with salt tolerance in plants has been sporadically reported. Lin et al. (2005) found that the Na^+^/H^+^ transporter NHE7 in mammals interacts with SCAMP2. SCAMP1 and SCAMP5 have also been shown to interact with NHE7 [20]. However, the direct role of the SCAMP protein family in soybean under abiotic stress has not been confirmed. This study investigates the overexpression of *GmSCAMP5* in soybean hairy root and its effect on salt tolerance. These results shown that *GmSCAMP5* plays a positive regulatory role in soybean’s salt tolerance. It is noteworthy that *GmSCAMP5* plays a crucial role in the transport of Na^+^ and K^+^, preventing the transport of Na^+^ from the root to the leaves and reducing salt damage in plants. Previous studies have demonstrated the importance of *GmNHX1*, *GmCLC1*, *GmTIP1*, *GmSOS1*, and *GmSOD1* in improving salt tolerance. *GmNHX1*, *GmCLC1*, *GmTIP1*, and *GmSOS1* are involved in ion transport, while *GmSOD1* is involved in the first line of defense against reactive oxygen species (ROS) [29–34]. The results of this study showed that these five genes were significantly upregulated in *GmSCAMP5*-OE plants under salt stress, suggesting that *GmSCAMP5* can regulate the expression of these genes in response to salt stress. However, the molecular mechanism of *GmSCAMP5* in soybean salt tolerance requires further research.

## Materials and methods

### Identification of *SCAMP* genes in soybean

GmSCAMP protein sequences were obtained from Phytozome (https://phytozome.jgi.doe.gov/pz/portal.html). SCAMP domain (PF04144) from the Pfam protein family database (https://pfam.xfam.org/) was used to search for predicted SCAMP proteins in soybean genome (G.max Wm82.a2.v1) with an e-value cut-off of 10^− 4^. The soybean SCAMP protein sequences were aligned using the HMM model in HMMERv3.0. The putative SCAMP gene core sequences were verified by performing searches against the Pfam (http://pfam.xfam.org/), NCBI-CDD (https://www.ncbi.nlm.nih.gov/cdd/) and SMART (http://smart.embl.de/) databases to confirm the presence of the SCAMP conserved domain, and redundant sequences manually. Visualize SCAMP conserved domains using IBS (http://ibs.biocuckoo.org/). The same methodology was used for identifying SCAMP genes in *Arabidopsis*, maize and rice. Physicochemical properties (number of amino acid, molecular weight, isoelectric point information, hydrophilia) for GmSCAMPs were obtained from ExPASy (http://web.expasy.org/protparam/). Transmembrane domain analysis was performed using Tmpred (http://www.ch.embnet.org/software/TMPRED form.html).

### Chromosomal location analysis

The physical locations of *SCAMP* genes on soybean chromosomes were extracted from the soybean genomic database. A physical location map was drawn by TBtools software.

### Phylogenetic analysis

The domain amino acid sequences of SCAMP proteins from soybean, *Arabidopsis*, maize, and rice were aligned by Clustal W. The phylogenetic tree was constructed and was rooted by using the human SCAMP2 as the outgroup using the NJ method in MEGA 7.0 [35] with 1,000 bootstrap replications.

### Analysis of gene structure and the conserved motif

The intron insertion sites in the *SCAMP* genes were identified by comparing the coding sequence with the corresponding gene full-length sequence using the GSDS (http://gsdscbi.pku.edu.cn/). The conserved SCAMP motifs were analyzed using the MEME5.2 (http://meme-suite.org/tools/meme); the maximum number of motifs was set to 15 [36].

### Promoter sequence analysis

The promoter sequences (1,500 bp upstream) of the *GmSCAMP*s were obtained from the Phytozome database and analyzed using Plant CARE database (http://bioinformatics.psb.ugent.be/webtools/plantcare/html/) [37]. Promoter graphs were drawn using GSDS and TBtools.

### Expression patterns of *SCAMP* Genes

The RNA-seq data for soybean *SCAMP* genes in various tissues at different developmental stages under normal conditions [38, 39], as well as their expression in the leaf, root and root hair under NaCl stress [40, 41], were extracted from NCBI, the raw sequence data in the form of fastq file of bio-projects PRJNA140081, PRJNA432861, PRJNA373634(GmSalinity-c), PRJNA373633(GmSalinity-b), PRJNA373632(GmSalinity-a), PRJNA373617(GmCtrl-1a), PRJNA373618(GmCtrl-1b), PRJNA373619(GmCtrl-1c) were used for the expression analysis. The log2(TPM + 1) was used to calculate the data of various tissues at different developmental stages under normal conditions, and the expression levels of *GmSCAMPs* were visualized using TBtools software.

### Plant materials and cultural condition

The plant materials used for gene expression pattern analysis were the salt tolerance variety ‘Fendou105’ and the salt sensitive variety ‘Shaanxibayuehuang’ (provided by Shanxi Agricultural University) [24]. The plant material Williams 82 was used for gene cloning and *Agrobacterium rhizogenes*-mediated transformation of soybean hairy roots.

The selected healthy seeds, which were free from pests and diseases, were soaked in a 0.1% sodium hypochlorite solution for 15 min. They were then rinsed with sterile water four to five times and spread on moist gauze for germination over a period of four days. Soybean buds exhibiting similar growth were chosen and transplanted into Hoagland nutrient solution in an artificial climate chamber (maintained at 25℃ with a light cycle of 14 h on and 10 h off). After approximately 14 days (once the third compound leaf was fully expanded), the soybean plants were exposed to a 150 mM NaCl solution for varying durations: 0, 1, 3, 6, 12, 24 and 48 h. Following the treatment, the roots, stems, and leaves were immediately submerged in liquid nitrogen and stored at -80℃ for subsequent qRT-PCR analysis.

### RNA extraction and gene cloning

Total RNA was isolated from distinct tissues of ‘Fendou105’ and ‘Shaanxibayuehuang’, as well as leaf of Williams 82, using Trizol according to the manufacturer’s protocol (TaKaRa). The cDNA was synthesized using the TransScript ® One-Step gDNA Removal and cDNA Synthesis SuperMix (TransGen) as manufacturer’s instructions. *GmSCAMP5* sequence was downloaded from Phytozome and used as a reference to clone *GmSCAMP5*. The primers were designed using Primer Premier 6.0. The primers sequences are listed in Supplementary Table S1, and synthesized by Sangon biotech (Shanghai) company.

### Quantitative real-time PCR (qRT-PCR) analysis

Three biological replicates were applied for quantitative real-time PCR (qRT-PCR) analysis. The relative expression levels of test genes were analyzed by the2^­ΔΔCt^methods [42]. The primers sequences are listed in Supplementary Table S1, and synthesized by Sangon biotech (Shanghai) company.

### Plasmid construction

For the construction of Gateway entry vectors, BP Clonase was used with pDONR207 and the purified PCR product, which were then transferred into *E. coli DH5α* after the PCR reaction. Similarly, LR Clonase was used with the overexpressing vector pB7WG2D and positive donor plasmids, as well as the RNA interference vector pK7WG2D and positive plasmids. The recombinant plasmids pB7WG2D-*GmSCAMP5* and pK7WG2D-*GmSCAMP5* were confirmed by sequencing. Recombinant plasmids pB7WG2D-*GmSCAMP5* and pK7WG2D-*GmSCAMP5*, along with the empty vectors pB7WG2D and pK7WG2D, were transformed into soybean using Agrobacterium (*A. rhizogenes* K599)-mediated genetic transformation.

### Evaluation of the tolerance agrobacterium rhizogenes-mediated transformation of soybean hairy roots to salt stress

Seeds of Williams 82 were surface-sterilized and germinated in sterile B5 medium (pH = 5.7) at 25℃ under a 16-hour (light) / 8-hour (dark) photoperiod for 3–4 days, until the cotyledons were about to open. The cotyledons were then carefully incised using sterile scalpels and co-cultivated with A. rhizogenes K599 (containing pB7WG2D-GmSCAMP2, pK7WG2D-GmSCAMP2, pB7WG2D, or pK7WG2D) on a petri dish lined with sterilized filter paper. This co-cultivation process took place at 28℃ in the dark for another 3–4 days. Afterwards, the soybean seedlings were transferred to vermiculite with Hoagland nutrient solution and placed under high humidity at 28◦C in the dark for approximately 3 days. They were then exposed to a 12-hour (light) / 12-hour (dark) photoperiod until hairy roots had developed at the infection site and reached a length of about 5 cm. Positive transgenic soybean hairy roots were identified using a handheld lamp (LUYOR-3415RG, USA), to detect green fluorescence signals. The soybean cotyledons containing positive hairy roots were then transferred to vermiculite at 25℃ for a duration of 7 days.

These plants were subjected to 0 mM (water control) and 200 mM NaCl for a period of 14 days. The levels of Na^+^ and K^+^ contents were measured according to the method described by Liu et al. (2016) [43]. The SPAD value in the leaf was measured by Chlorophyll Meter (SPAD-502Plus, Japan). To assess the effects of NaCl treatment, the leaves were soaked in a 0.5% trypan blue solution (Coolaber, China) for 12 h, followed by decoloration in 75% ethanol. This process was carried out in triplicate, with three independent transgenic plants measured for each repetition.

Furthermore, RNA was isolated from more than 10 randomly selected positive soybean hairy roots, and cDNA was synthesized for detecting gene expression. Five genes were selected for analysis of the mechanism of *GmSCAMP5*-mediated salt resistance in soybean, which were reported to play an important role in salt stress. *GmNHX1*(Glyma.20g229900), a Na^+^/H^+^ antiporter gene, localized in the tonoplast could sequester ions (especially Na^+^) from cytoplasm into vacuole to reduce its toxic effects [29]. *GmCLC1*(*Glyma.05g077100*), a chloride channel gene, enhanced salt tolerance by reducing Cl^−^ accumulation to reduce the negative impact of salt stress [30]. *GmTIP1*(*Glyma.03g185900*), a tonoplast intrinsic proteins gene, confers salt tolerance to soybean [31]. *GmSOS1*(*Glyma.08g092000*), a salt overly sensitive gene, characterized Na^+^ efflux transporter, plasma membrane Na^+^/H^+^ exchanger plays a critical role in soybean salt tolerance by maintaining Na^+^ homeostasis [32]. *GmSOD1*(*Glyma.19g240400*), a superoxide dismutase gene, functions as a first line of defense against reactive oxygen species (ROS) [33], transcript accumulation of *GmSOD1* to overcome heat and salinity induced oxidative damage [34].

### Electronic supplementary material

Below is the link to the electronic supplementary material.

**Supplementary Material 1: Figure S1.** Analysis of SCAMP conserved domains. NCBI-CDD (https://www.ncbi.nlm.nih.gov/cdd/) databases to confirm the presence of the SCAMP conserved domain, and visualize SCAMP conserved domains using IBS (http://ibs.biocuckoo.org/). Differently colored boxes indicate different types of domains, and red depicts the SCAMP conserved domain. Digital indicated the position of the SCAMP domain in the soybean SCAMP genes. **Figure S2.** Chromosomal distribution of *GmSCAMPs* gene. 10 *GmSCAMPs* were identified from soybean genome and named *GmSCAMP1* to *GmSCAMP10* according to their distribution positions on chromosomes. **Figure S3.** Gene structure of *GmSCAMPs*. The exon and intron structures of the 10 *GmSCAMP* genes were compared. **Figure S4.** Conserved domains of *GmSCAMPs*. Conservation of motifs in the 10 GmSCAMP proteins was identified using the MEME website. (A) 10 different conserved motifs were indicated by colored box, namely motif1-10. (B) motif logo in the 10 GmSCAMP proteins was identified, A: Alanine, C: Cysteine, D: Aspartic acid, E: Glutamic acid, F: Phenylalanine, G: Glycine, H: Histidine, I: Isoleucine, K: Lysine, L: Leucine, M: Methionine, N: Asparagine, P: Proline, Q: Glutamine, R: Arginine, S: Serine, T: Threonine, V: Valine, W: Tryptophan, Y: Tyrosine. **Figure S5.** Expression profiles of *GmSCAMP* genes in different soybean tissues. The expression abundance of each transcript is represented by the color: red, higher expression; blue, lower expression. Expression levels in the whole seeds at five stages of seed development (globular, heart, cotyledon, early-maturation, mid-maturation, late-maturation, dry), and vegetative (leaves, roots, stems, seedlings) and reproductive (floral buds) tissues. **Figure S6.** Fluorescence detection of positive hairy roots. Fluorescence detection of positive hairy roots in *SCAMP5*-RNAi, EV-Control, and *SCAMP5*-OE. **Table S1.** Physical and chemical properties of GmSCAMPs. **Table S2.** Primers used in this study

## Data Availability

The RNA-seq data used in this study were download from NCBI Database, Sequence data from this article can be found in the GenBank/EMBL or Glycine max Wm82.a4.v1 database with the following entry number: *GmSCAMP1* (*Glyma.01g010300*), *GmSCAMP2* (*Glyma.06g296900*), *GmSCAMP3* (*Glyma.07g191600*), *GmSCAMP4* (*Glyma.09g210800*), *GmSCAMP5* (*Glyma.12g108200*), *GmSCAMP6* (*Glyma.12g202000*), *GmSCAMP7* (*Glyma.13g185100*), *GmSCAMP8*(*Glyma.13g244600*), *GmSCAMP9*(*Glyma.13g185100*), *GmSCAMP10* (*Glyma.13g185100*).
