# Supplementary material for: Genome-wide analysis of the SCAMPs gene family of soybean and functional identification of GmSCAMP5 in salt tolerance
Source: BMC Plant Biol. 2023 Dec 8;23:628. doi: 10.1186/s12870-023-04649-2 (PMC10704743; doi:10.1186/s12870-023-04649-2)
Supplement: Supplementary file 1 — Supplementary Material 1: Figure S1. Analysis of SCAMP conserved domains. NCBI-CDD (https://www.ncbi.nlm.nih.gov/cdd/) databases to confirm the presence of the SCAMP conserved domain, and visualize SCAMP conserved domains using IBS (http://ibs.biocuckoo.org/). Differently colored boxes indicate different types of domains, and red depicts the SCAMP conserved domain. Digital indicated the position of the SCAMP domain in the soybean SCAMP genes. Figure S2. Chromosomal distribution of GmSCAMPs gene. 10 GmSCAMPs were identified from soybean genome and named GmSCAMP1 to GmSCAMP10 according to their distribution positions on chromosomes. Figure S3. Gene structure of GmSCAMPs. The exon and intron structures of the 10 GmSCAMP genes were compared. Figure S4. Conserved domains of GmSCAMPs. Conservation of motifs in the 10 GmSCAMP proteins was identified using the MEME website. (A) 10 different conserved motifs were indicated by colored box, namely motif1-10. (B) motif logo in the 10 GmSCAMP proteins was identified, A: Alanine, C: Cysteine, D: Aspartic acid, E: Glutamic acid, F: Phenylalanine, G: Glycine, H: Histidine, I: Isoleucine, K: Lysine, L: Leucine, M: Methionine, N: Asparagine, P: Proline, Q: Glutamine, R: Arginine, S: Serine, T: Threonine, V: Valine, W: Tryptophan, Y: Tyrosine. Figure S5. Expression profiles of GmSCAMP genes in different soybean tissues. The expression abundance of each transcript is represented by the color: red, higher expression; blue, lower expression. Expression levels in the whole seeds at five stages of seed development (globular, heart, cotyledon, early-maturation, mid-maturation, late-maturation, dry), and vegetative (leaves, roots, stems, seedlings) and reproductive (floral buds) tissues. Figure S6. Fluorescence detection of positive hairy roots. Fluorescence detection of positive hairy roots in SCAMP5-RNAi, EV-Control, and SCAMP5-OE. Table S1. Physical and chemical properties of GmSCAMPs. Table S2. Primers used in this study [file 12870_2023_4649_MOESM1_ESM.docx]

**Supplementary information**

**Figure S1** Analysis of SCAMP conserved domains. NCBI-CDD ([https://www.ncbi.nlm.nih.gov/cdd/](https://www.ncbi.nlm.nih.gov/cdd/)) databases to confirm the presence of the SCAMP conserved domain, and visualize SCAMP conserved domains using IBS (<http://ibs.biocuckoo.org/>). Differently colored boxes indicate different types of domains, and red depicts the SCAMP conserved domain. Digital indicated the position of the SCAMP domain in the soybean SCAMP genes.

**Figure S2** Chromosomal distribution of *GmSCAMPs* genes.10 *GmSCAMPs* were identified from soybean genome and named *GmSCAMP1* to *GmSCAMP10* according to their distribution positions on chromosomes.

**Figure S3** Gene structure of *GmSCAMPs.* The exon and intron structures of the 10 *GmSCAMP* genes were compared.

**Figure S4** Conserved domains of *GmSCAMPs.* Conservation of motifs in the 10 GmSCAMP proteins was identified using the MEME website. (A)10 different conserved motifs were indicated by colored box, namely motif1-10. (B) motif logo in the 10 GmSCAMP proteins was identified, A:Alanine, C: Cysteine, D: Aspartic acid, E: Glutamic acid, F: Phenylalanine, G: Glycine, H: Histidine, I:Isoleucine, K:Lysine, L: Leucine, M: Methionine, N: Asparagine, P: Proline, Q: Glutamine, R: Arginine, S: Serine, T: Threonine, V: Valine, W: Tryptophan, Y:Tyrosine.

**Figure S5** Expression profiles of *GmSCAMP* genes in different soybean tissues. The expression abundance of each transcript is represented by the color: red, higher expression; blue, lower expression. Expression levels in the whole seeds at five stages of seed development (globular, heart, cotyledon, early-maturation, mid-maturation, late-maturation, dry), and vegetative (leaves, roots, stems, seedlings) and reproductive (floral buds) tissues.

**Figure S6** Fluorescence detection of positive hairy roots. Fluorescence detection of positive hairy roots in SCAMP5-RNAi, EV-Control, and SCAMP-OE.

**Table S1** Physical and chemical properties of GmSCAMPs.

**Table S2** Primers used in this study.


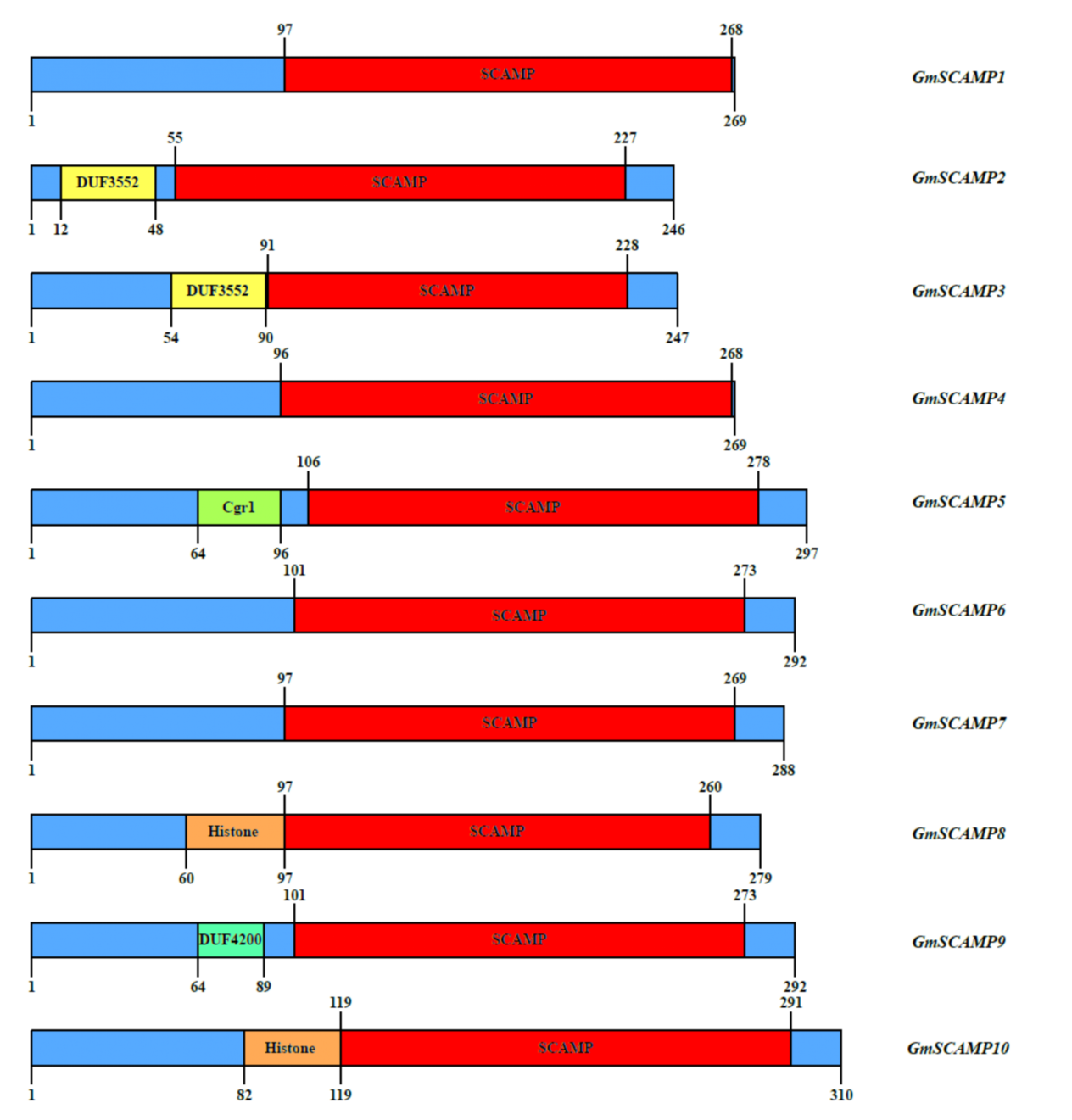


**Figure S1** SCAMP domains analysis in soybean SCAMP family members.


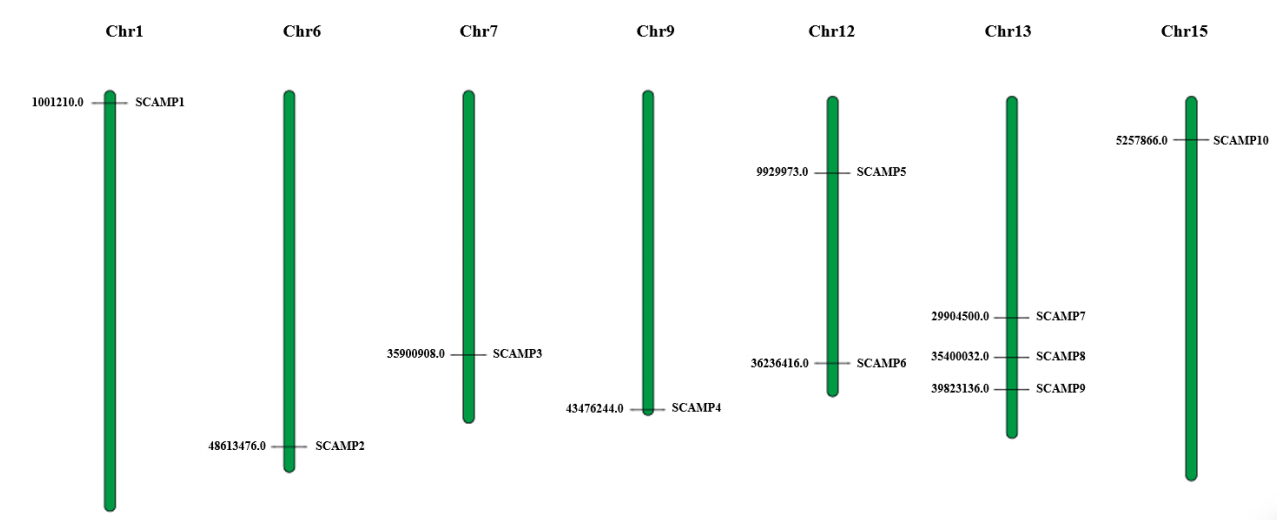


**Figure S2** Chromosomal distribution of *GmSCAMPs* genes


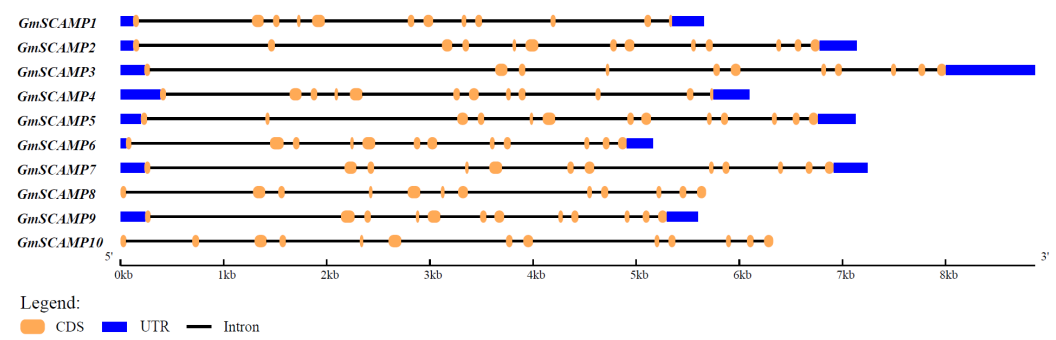


**Figure S3** Gene structure of *GmSCAMPs*


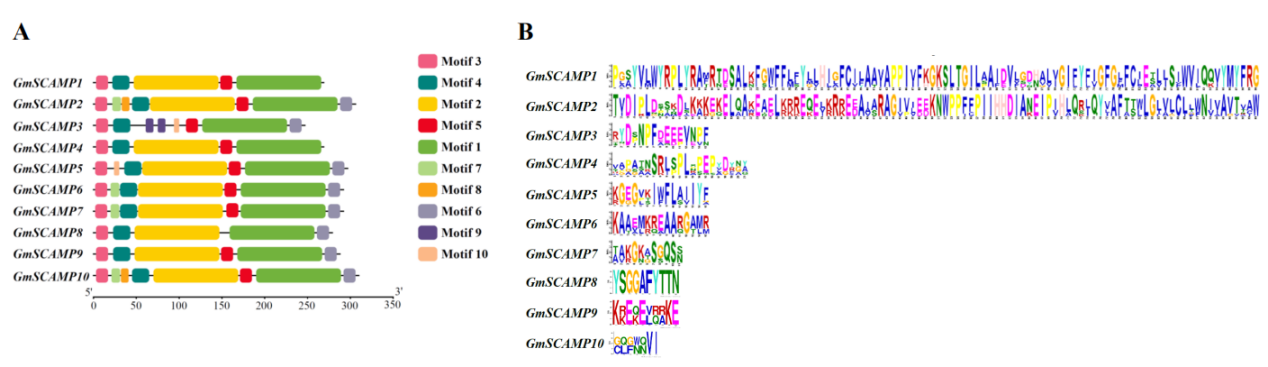


**Figure S4** Conserved domains of *GmSCAMPs*

*
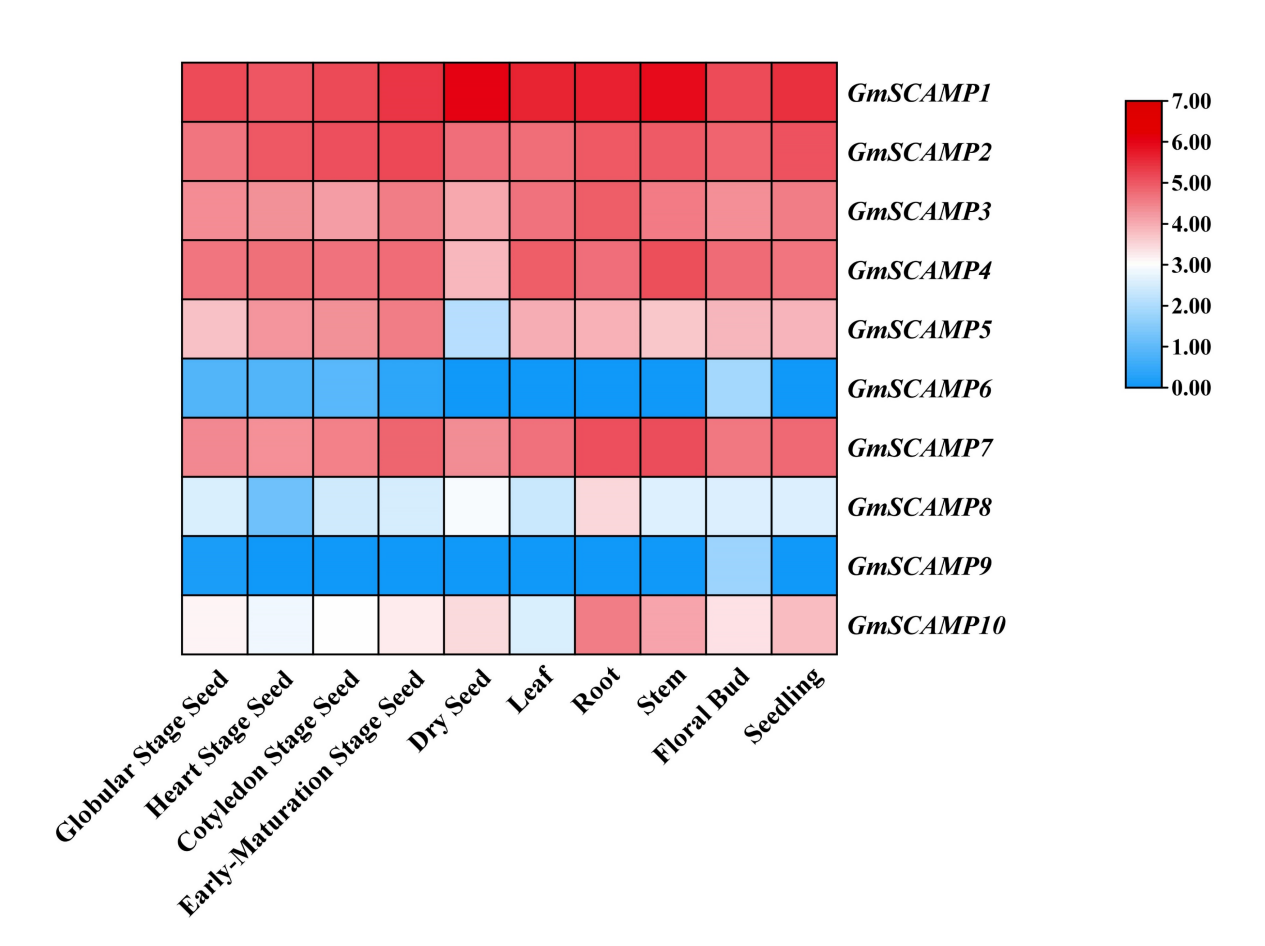
*

**Figure S5** Expression profiles of *GmSCAMP* genes in different soybean tissues.


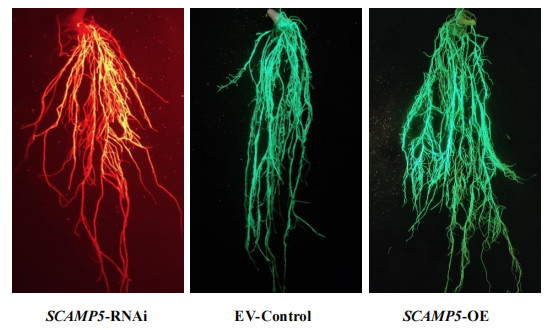


**Figure S6** Fluorescence detection of positive hairy roots.

**Table S1** Physical and chemical properties of *GmSCAMPs.*

| **Gene name** | **Gene ID** | **Number of amino acids** | **Molecular weight(Da)** | **Protein isoelectric point** | **hydropathicity** | **Transmembrane domain** |
| --- | --- | --- | --- | --- | --- | --- |
| *GmSCAMP1* | *Glyma.01g010300* | 254 | 29039.72 | 6.34 | 0.208 | 4 |
| *GmSCAMP2* | *Glyma.06g296900* | 306 | 34077.66 | 8.81 | 0.140 | 4 |
| *GmSCAMP3* | *Glyma.07g191600* | 247 | 27751.43 | 8.86 | 0.189 | 4 |
| *GmSCAMP4* | *Glyma.09g210800* | 269 | 30391.54 | 8.62 | 0.230 | 4 |
| *GmSCAMP5* | *Glyma.12g108200* | 297 | 33541.29 | 8.31 | 0.175 | 4 |
| *GmSCAMP6* | *Glyma.12g202000* | 292 | 32504.64 | 6.96 | 0.178 | 4 |
| *GmSCAMP7* | *Glyma.13g185100* | 288 | 32564.99 | 8.30 | 0.195 | 4 |
| *GmSCAMP8* | *Glyma.13g244600* | 279 | 31555.62 | 8.34 | 0.129 | 4 |
| *GmSCAMP9* | *Glyma.13g300100* | 292 | 32517.66 | 7.65 | 0.190 | 4 |
| *GmSCAMP10* | *Glyma.15g069100* | 310 | 34658.96 | 6.61 | 0.090 | 4 |

**Table S2** Primers used in this study.

| **Primers** | **Sequence（5'­­3'）** |
| --- | --- |
| *GmCYP2*- Forward | CGGGACCAGTGTGCTTCTTCA |
| *GmCYP2*- Reverse | CCCCTCCACTACAAAGGCTCG |
| *GmSCAMP5-*Qpcr-Forward | TTTTGGCTGCGATTGACGTG |
| *GmSCAMP5-*Qpcr-Reverse | TCTGGCAGCCTCACGTTTTA |
| *GmSCAMP5-*Forward | ATGAGTCGCTACGATCCCAATC |
| *GmSCAMP5-*Reverse | TCATAGAGCTGCCATCATTGTGC |
| *GmNHX1-* Forward | AAGCAGCATCCGTGCTTTAC |
| *GmNHX1-* Reverse | CCTGCCACCAAAAACAGGAC |
| *GmCLC1-* Forward | TTGTGGTGCTTCTTCAGGGG |
| *GmCLC1-* Reverse | AAGGCTTCCCAATACCCCAC |
| *GmTIP1-*Forward | TGTCTTCATCAGCCACACCC |
| *GmTIP1-*Reverse | AGATCAACGGCCACCGATTC |
| *GmSOD1-* Forward | GCTTCAGTATTACCGACAGTCA |
| *GmSOD1-* Reverse | CACAAGCTACTCTGCCACCA |
| *GmSOS1-* Forward | CACTGCTCAGGAGGGTTCAG |
| *GmSOS1-* Reverse | GAAAGCAAGAGGTGGGTCC |
